# Endometriosis of the uterine cesarean section scar: A case report

**DOI:** 10.4103/0971-3026.37111

**Published:** 2008-02

**Authors:** Ashim K Lahiri, Kiran Sharma, Naser Busiri

**Affiliations:** Department of Radiology, Farwania Hospital, P.O. Box - 18373, Kuwait - 81004; 1 Department of Obstetrics and Gynecology, Farwania Hospital, P.O. Box - 18373, Kuwait - 81004

**Keywords:** Cesarean scar, endometriosis, MRI

Endometriosis is defined as the presence of functional endometrial tissue outside the uterine cavity.[1,2] The most common sites of involvement, in decreasing order of frequency, are the ovaries, pelvic peritoneum, deep pelvic subperitoneal spaces, the intestinal system, and the urinary system.[1] Scar endometriosis is a rare disease which is difficult to diagnose.[3,4] The diagnosis is frequently made only after excision and histopathology of the lesion. Cases of scar endometriosis of the abdominal wall following various obstetrical and gynecological procedures have been reported.[2–4] However, endometriosis of a uterine scar is extremely rare.[2] Kafkasli *et al*.[2] after reviewing the pathology reports of hysterectomy specimens of the previous seven years, found only two cases of endometriosis in uterine wall Cesarean section scars. One of these two cases had adenomyosis of the uterus in addition to endometriosis of the uterine scar.

We report a case of endometriosis of a uterine wall scar, presenting as a mass in the anterior lower part of uterus.

## Case Report

A 42-year-old lady presented with a history of intermenstural bleeding and pelvic pain for 6 months. She had a history of two previous lower segment Cesarean sections (LSCS).

Clinically, the abdomen was soft and lax with a healthy scar of the previous LSCS. On per vaginal examination, the uterus was bulky, nontender, had an irregular outline, and was deviated to the left.

**Figure 1 (A, B):**
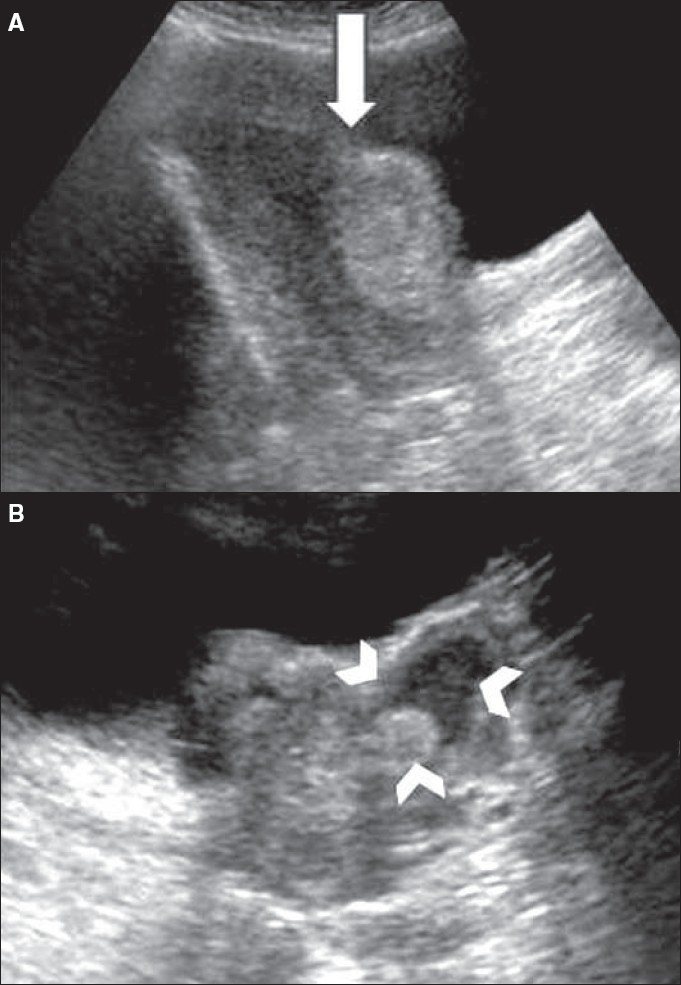
USG in the longitudinal (A) and transverse (B) planes shows the echogenic lower uterine mass (arrow), with contents of mixed echogenecity (arrowheads)

Transabdominal and transvaginal USG revealed a mildly bulky uterus with a heterogenous myometrial echotexture [Figure 1]. A lobulated, predominantly echogenic complex mass was seen arising from the lower uterine segment, measuring 85 × 32 × 38 mm, and extending outwards into the uterovesical region. The periphery of this mass was in continuation with the uterine wall. The central part of the mass showed hypoechoic and heterogeneous hyperechoic areas communicating with the lower uterine cavity. The uterine body endometrium was of normal thickness. The ovaries were of normal size and no adnexal masses were seen. No likely diagnosis could be made.

**Figure 2:**
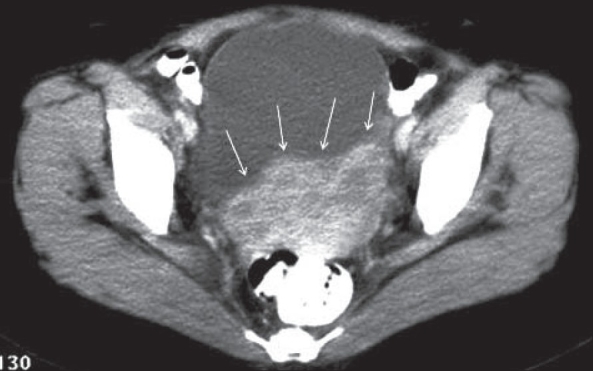
Axial CT image through the mass shows heterogeneously enhancing nodular masses (arrows)

**Figure 3:**
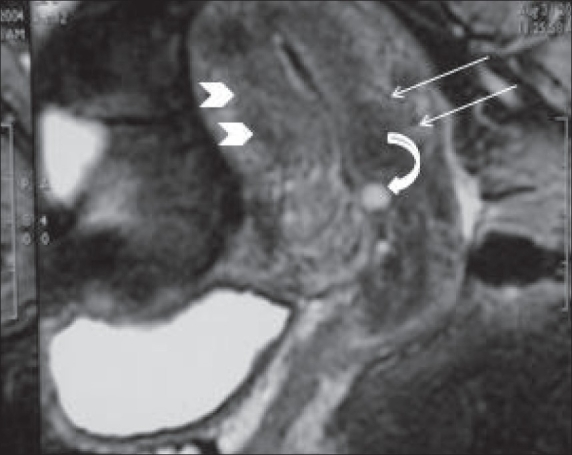
Sagittal T2W MRI of the uterus shows features of adenomyosis (more marked in the lower uterine segment) with thickened junctional zone of the myometrium (arrowheads) and a small hyperintense myometrial cyst (curved arrow)

**Figure 4 (A, B):**
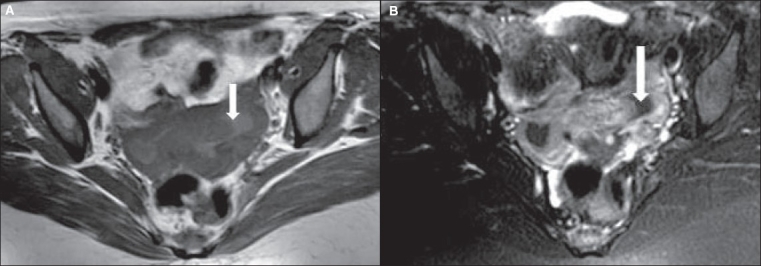
Axial MRI of the uterus shows the contents of the mass to be of high signal intensity on T1W image (A) and of low signal intensity on T2W image (B), consistent with blood products

**Figure 5:**
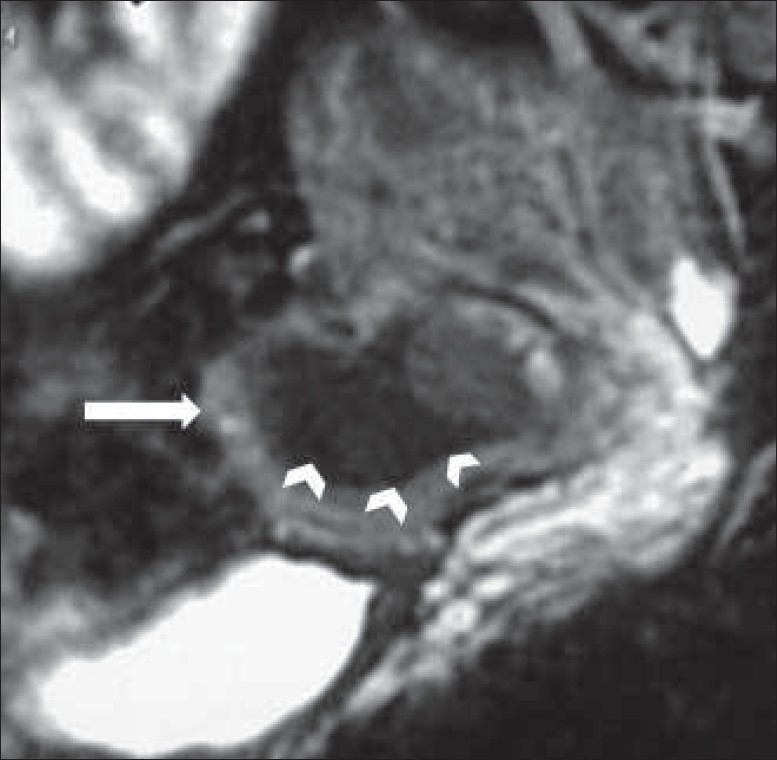
Sagittal T2W MRI image shows the anterior lower segment uterine mass (arrow) with the presence of blood products (arrowheads)

A contrast-enhanced CT scan [Figure 2] showed heterogeneously enhancing, nodular masses arising from the lower uterine region. The possibility of fibroids was considered. MRI [Figures 3–5] done subsequently showed a lobulated mass projecting outwards from the anterior lower uterine wall into the uterovesical space, but not invading the urinary bladder or any other adjacent pelvic organ. The signal characteristics of the wall of the mass were similar to that of the myometrium and the mass was in continuation with the uterine wall. The luminal contents of the mass were of high signal intensity on T1W images and low on T2W images, suggesting blood products. The junctional zone of the myometrium in the lower uterine segment was thickened (16 mm) with indistinct margins [Figure 4]. Small myometrial cysts were also seen. The ovaries were normal and separate from the mass. No peritoneal lesions or adnexal masses were seen. The MRI diagnosis was adenomyosis, with a possibility of associated endometriosis in the uterine wall.

After the radiological work-up the patient was subjected to diagnostic laparoscopy, which revealed uterine scar endometriosis. Multiple cystic masses were seen in the lower uterine segment, with oozing of chocolate brown material. Laparoscopy did not reveal any endometriotic deposits elsewhere in pelvis. As the mass was deeply embedded in the lower part of uterus, a laparotomy was decided upon and total abdominal hysterectomy was performed with conservation of both ovaries. The postoperative period was uneventful. Histopathology confirmed the diagnosis of endometriosis of the uterine scar; in addition, evidence of adenomyosis was seen, mostly in the anterior uterine wall.

## Discussion

Endometriosis is one of the commonest benign gynecological conditions; it is estimated to occur in 10% of the female population and occurs almost exclusively in women of reproductive age.[5] The endometriotic lesions are hormone dependant and tend to bleed with each menstrual cycle, becoming more congested and larger in size, with patients feeling cyclical pain and discomfort.[6] The most common sites of involvement, in decreasing order of frequency, are the ovaries, pelvic peritoneum, deep subperitoneal spaces, the intestinal system, and the urinary system.[1] Scar endometriosis is a rare disease entity.[3,4] Cases of abdominal wall scar endometriosis, previously reported in literature, have shown deposits in the dermal and subcutaneous tissue, the rectus abdominis muscle, and the rectus sheath.[2] The reported incidence of abdominal scar endometriosis following hysterotomy is 1.08-2%, whereas after Cesarean section the incidence is 0.03-0.4%.[2–4] However, endometriosis of the uterine wall scar is an extremely rare disease entity and no statistics are available regarding its incidence and prevalence.[2] The etiology of scar endometriosis has been attributed to the implantation of decidual cells during various surgical procedures, which subsequently proliferate or induce metaplasia in the surrounding cells under the influence of estrogen, to cause endometriosis.

USG is recommended for detecting endometriomas of the ovary, bladder, and rectum but it is less sensitive than MRI for assessment of deep pelvic endometriosis.[1,5,7] Similarly, CT also does not play any specific diagnostic role in these cases. On the other hand, MRI is extremely useful because of its very high spatial resolution, which enables accurate detection of very small hemorrhagic lesions.[5–7] The sensitivity and specificity of MRI in diagnosing endometriomas are very high, being 90-92% and 91-98%, respectively.[1,5,6] Furthermore, MRI is a useful modality for presurgical mapping of deep pelvic endometriosis.[1,7] Infiltration of abdominal wall muscles and subcutaneous tissues is much better assessed by MRI.

MRI proved extremely helpful for diagnosing endometriosis in this case because the MR signal characteristics on various sequences indicated the presence of blood products within the uterine lesion. In addition, findings diagnostic of adenomyosis were also seen in our case.[8] The involvement of the lower segment uterine Cesarean scar was not considered preoperatively. The histopathology confirmed endometriosis of the Cesarean scar and, in addition, adenomyosis of the anterior uterine wall was also reported. Occurrence of adenomyosis along with endometriosis is well known, and Kafkasli *et al*[2] had also reported a similar finding in their case report. In this case, no ovarian endometriomas or peritoneal lesions were identified by imaging, per-operatively, or histopathologically.
